# Low intake of commonly available fruits and vegetables in socio-economically disadvantaged communities of South Africa: influence of affordability and sugary drinks intake

**DOI:** 10.1186/s12889-019-7254-7

**Published:** 2019-07-12

**Authors:** Kufre Joseph Okop, Kululwa Ndayi, Lungiswa Tsolekile, David Sanders, Thandi Puoane

**Affiliations:** School of Public Health, University of the Western Cape, Private Bag X17, Bellville, 7535 South Africa

**Keywords:** Fruit and vegetables, Daily, Intake, Affordability, South Africa, Community

## Abstract

**Background:**

Consumption of fruits and vegetables reduces the risk of obesity, diabetes, cancer, cardiovascular mortality and all-cause mortality. The study assessed the pattern of intake and the factors that influence daily intake of commonly available fruits and vegetables in economically disadvantaged South African communities.

**Methods:**

This is a cross-sectional study nested on an ongoing longitudinal study in South Africa. Two communities (a rural and urban) of low socio-economic status were purposely selected from two of the nine provinces. A sample of 535 participants aged 30–75 years was randomly selected from the longitudinal cohort of 1220; 411 (78%) women. Data were collected using validated food frequency and structured interviewer-administered questionnaires. Descriptive and multivariate regression analysis were undertaken.

**Results:**

A higher proportion of participants in the urban township compared to their rural community counterparts had purchased fruits (93% vs. 51%) and vegetables (62% vs. 56%) either daily or weekly. Only 37.8% of the participants consumed at least two portions of commonly available fruits and vegetables daily, with no differences in the two communities. Daily/weekly purchase of sugar sweetened beverages (SSBs) was associated with daily intake of fruits and vegetables (*p* = 0.014). Controlling for age and gender, analysis showed that those who spent R1000 (USD71.4) and more on groceries monthly compared to those who spent less, and those who travelled with a personal vehicle to purchase groceries (compared to those who took public transport) were respectively 1.6 times (AOR, 95% CI: 1.05–2.44; *p* = 0.030) and 2.1 times (AOR, 95% CI: 1.06–4.09; *p* = 0.003) more likely to consume at least two or more portions of fruits and vegetables daily. Those who purchased SSBs daily or weekly were less likely (AOR, 95% CI: 0.54, 0.36–0.81, *p* = 0.007) to consume two or more portions of fruits and vegetables daily. The average household monthly income was very low (only 2.6% of households earned R5000 (US$357.1); and education level, attitude towards fruits and vegetables and owning a refrigerator had no significant association with fruits and vegetable daily intake.

**Conclusion:**

These findings indicate that affordability and frequency of purchase of sugary drinks can influence daily intake of fruits and vegetables in resource-limited communities.

## Background

The prevalence of consumption of fruits and vegetables below the recommended daily intake is a persistent phenomenon in many developing countries [1–3]. Nearly 3.0% (approximately 1.7 million) of global deaths are attributable to low fruits and vegetable consumption. Insufficient intake of fruits and vegetables results in about 14% of gastrointestinal cancer deaths, 11% of ischaemic heart disease deaths and 9% of stroke deaths [4, 5]. In South Africa, a comparative burden of disease study reported that low fruits and vegetable intake accounted for 3.2% (1,667) of 521,000 deaths and a 1.1% disability-adjusted life years (DALY) [6].

Adequate intake of fruits and vegetables is considered an essential option for disease prevention and maintaining optimal health [7, 8]. Increasing evidence shows that the consumption of fruits and vegetables prevent weight gain, and reduces the incidence of type 2 diabetes, and the risk of cancer, certain eye diseases, dementia and osteoporosis [9, 10]. In a recent multi-country Prospective Urban and Rural Epidemiology (PURE) study involving low-, middle-, and high-income countries, consumption of higher portions of fruits and vegetables (seven portions and above) indicated a reduced risk for cancer (0.75 (0.59–0.96) and cardiovascular mortality (0.69 (95% CI: 0.53–0.88)) [3]. An increased proportion of fruits and vegetable consumption is also linked to a decrease in all-cause mortality [11].

According to the World Health Organization reports, eating at least five portions of fruits and vegetables (a recommended minimum of 400 g) per day reduces the risk of non-communicable diseases (NCDs) and also ensures an adequate daily intake of dietary fibre [4, 5]. However, studies showed that consumption of these foods in the recommended amounts is very low in poor populations unlike in Europe and the USA [12]. The ‘at-least-five-portions’ recommendation for fruits and vegetables intake is largely based on observational data from Europe and the USA, and therefore, has not been feasible in many resource-poor and economically disadvantaged settings [12, 13]. A multi-country study by Hall et al. reported that 77.6% of men and 78.4% of women (based on findings from 52 low- and middle-income countries) consumed less than the minimum recommended five daily servings of fruits and vegetables [14]. The South African National Health and Nutrition Survey 2013 (SANHANES-1), for an example, reported a very low intake of fruits and vegetables among South Africans [15]. This situation is probably due in part to the socioeconomic deprivation resulting high proportion of unemployment, and lack of income and limiting choice of diet in the population [3].

Studies on food prices and diet cost had pointed to the socio-economic disparity in dietary intake and health [16]. A systematic review and meta-analysis have also reported that consumption of acceptable healthier food (example, fruits and vegetables) in many studies are commonly associated with higher costs, disproportionally high in low-income settings [16, 17]^.^ In many developing countries, inadequate consumption of fruits and vegetables have been documented with substantial variability by country and socioeconomic status [14, 18]^,^.

In a study conducted in seven African countries in 2010, a considerable proportion of school boys and girls consumed less than one fruit (36 and 33%) and vegetable (23 and 22%) per day [19]. A large multi-country study involving South African cohorts recently reported a low consumption of healthy food particularly, fruits and vegetables, and that this decreased with increasing cost [3]. There is generally a disproportionately high level of sugar-sweetened beverages (SSBs) intake reported among the poor food insecure adults, which is believed to result (perhaps, in part) in a decline in intake of fruits and vegetables in the poor settings of South Africa [20–22].

Although South Africa is undergoing nutrition and epidemiological transitions, research relating to the food environment, access, purchase and consumption patterns of healthy diets, particularly, fruits and vegetables in resource-poor settings is limited. Moreover, the findings from the food-based dietary surveys in South Africa have shown that the recommended 5-servings guideline (i.e. 2 servings of fruits and 3-servings of vegetables/day) has not been a norm in economically disadvantaged communities [23, 24]. The national survey (SANHANES-1, 2014 version) further showed that only 4.6% of the adults consumed four or more fruits per day while the majority (52.2%) reported consuming one to three fruits per day (irrespective of portion size) [25]. This situation is similar in other low-middle income settings, as a recent study in Tanzaina, reported that 82% of the participants aged ≥15 years did not meet the recommended daily fruits and vegetables intake, and only 16 and 44% had consumed fruits and vegetables daily, respectively [26].

Furthermore, the SANHANES-1 study, reported that 25.6% of South Africans had low score (i.e.0–2 fruits/day) for daily fruits and vegetables intake. This low intake has been linked to the high cost and unavailability in poor communities [15]. In the present study, we assessed the frequency and pattern of intake of commonly available fruits and vegetables in resource-poor South African communities, and the possible factors associated with daily intake of at least two portions.

## Methods

### Design and setting

This study is a cross-sectional study nested on an ongoing longitudinal study. Two communities, namely, an urban township (Langa) near Cape Town metropolis and a rural community (Mt Frere) in the Eastern Cape Province involved in the ongoing Prospective Urban and Rural Epidemiology (PURE) study were selected using a two-stage sampling described previously [27]. These two communities are regarded as economically disadvantaged communities based on their socioeconomic status (SES) [28]. Langa is a black African township near Cape Town which has grown because of migration of persons mostly from the rural Eastern Cape. It is reported that most residents live with an average monthly household income of R2,144 ($200) and over 40% were unemployed as of 2015 [28, 29]. Generally, the Langa community has been grouped into three development areas namely, “old Langa”, “the Zones” and “the Hostels” which mirror the SES of the residents. The second study community, Mount Frere is a rural community located in Alfred Nzo district in the Eastern Cape with an estimated 99.8% black African and an estimated population density of 519 km^2^. Most residents earn an average monthly income between R1001–2500 ($80–$200); with an estimated unemployment rate of over 76% [28]. This study is an aspect of the collaborative research of the Centre of Excellence on Food Security (CoEFS) in the University of Western Cape. The intent of the CoEFS study was to explore food security, lifestyle and health status in the poor commnties and to use the information to support the implementation of interventions on lifestyle modification in similar populations.

### Sample size and sampling methods

**Fig. 1:**
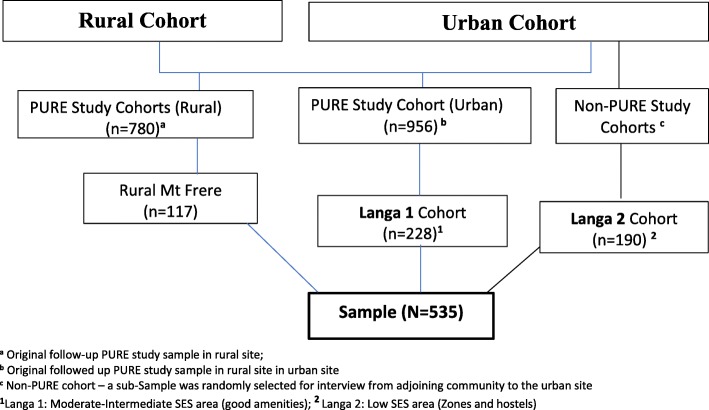
Sampling frame

The national survey (SANHANES-1) in 2012 reported that 25.6% of South Africans have a low dietary intake score (i.e. ≤ 2 out of 8) for fruit and vegetables, with 28.3% low intake score among black adults [15]. Based on an approximated 29% proportion of the outcome in the study population, we calculated a sample size of 535 considering a 5% level of precision, 1.5 design effect (for two stage sampling) and after adjustment (12%) for non-response rate and differences by sex. The 12% non-response rate was justified by our previous study in this population, for which nearly 12% of persons visited were not willing to participate in the study for some reasons [30]. Sample size calculations were performed with Epi Info. A priori, the decision was to recruit 70% of adults age 30–75 years from the two PURE study cohorts and the remainder from the non-PURE sub-sample in an adjoining low SES area of the urban site. Random sampling was used to select the participants from the urban township and rural, and a sub-sample from the urban township – as depicted in Fig. 1. Most of those recruited (345, 66%) were from the rural and urban PURE study cohorts, and the remainder (190, 34%) were sampled from an adjoining low SES area (the ‘Zones’ and ‘hostels’) - Langa 2.

For the current study, we first sub-divided the urban township into two areas: Langa 1 and Langa 2 which mirrors the SES of the three different sections of the community described previously [31]. Langa 1 (old Langa) is considered as the moderate-high SES area, with better established social amenities, whereas the Langa 2 (the zones and hostels) is classified as low SES area with fewer amenities. The rural study community (Mt Frere) was also classified as low SES area. A systematic random sampling of every second household in each of the two SES areas in the urban township was undertaken. Households included were those with at least one member who was between 35 and 70 years old. Trained field workers approached eligible individuals in the study sites households for recruitment. In the rural community, cluster sampling was used to sample the eligible household members. The non-PURE study participants were sampled from every second adjoining community to the PURE-study in the urban site.

### Data collection and analyses

A list of available and commonly consumed fruits and vegetables at the time of the study were obtained from the food frequency questionnaire that was used for collecting data from the PURE study participants, and also for the non-PURE participants [3]. The participants were asked about their food purchases and intake patterns including the frequency of fruits and vegetable intake, meat, snacks and sugar-sweetened beverages. Specifically, each participant was asked, “*How often (daily, twice weekly, weekly, monthly/seldom) do you eat a portion of each of the listed commonly consumed fruits and vegetables in a typical month?”*. A portion of fruit was provisionally defined as one half to 1 large/small size of commonly available fruits, and a portion of vegetable, as one half to one cup of the listed vegetables (green leafy, dried, or cooked or canned). Questions on household income, grocery expenditure, food choices, transport choice, and socio-demographics were obtained to describe determinants of fruits and vegetable consumption. The information was collected using a previously piloted structured 51-item interviewer-administered questionnaire. Information on health status and risk factors of the participants were collected from the main (PURE) study questionnaire, to determine the factors that are associated with daily intake of the commonly available fruits and vegetables in the study. Data were collected between August and December 2015. For the objective of this paper, we focus on the daily intake of the fruits and vegetables that participants had considered available or in season at the time of the study. Preliminary analysis had shown that less than 30% of the study participants in this cohort self-reported consuming less than two servings of fruits or vegetables in a day. For this, we considered self-reported daily intake of at least two portions of the listed commonly available fruits (orange, apple, pear, banana, peach, watermelon, tomatoes, mango and grapes) and vegetables (spinach, cabbage, pumpkin, carrot, green pea, green leafy vegetables, and onions) as proxy of the servings of fruits and vegetable consumed per day.

Descriptive analysis was employed to profile the study participants’ characteristics by gender. Pearson Chi-square tests and ANOVA (mean) comparison were used to describe and compare intake patterns by communities, SES areas and NCD morbidity. Multivariate logistic regression analysis was undertaken to determine the factors associated with daily intake of commonly available fruits and vegetables. In order to fit an appropriate model, we had considered, first of all to explore the relationships of the outcome variable with the explanatory variables, individually. Then, we got a clue to what variables might be important to fit the multivariable model to answer our research question. Data were analysed using SPSS version 24, and the level of significance was taken as *p* < 0.05.

## Results

### The participants’ characteristics

The participants’ characteristics by SES area are presented in Table 1. There were significant differences in the demographic characteristics (except for gender) and household assets by SES area. Most of the study participants in the SES areas were women (76.8%). About half (51%) of participants were younger than 50 years old. A greater percentage (67%) had more than primary school education, 77% were either unemployed, with the highest proportion of unemployment in the rural study site. More than half (55%) reported a household income of less than R2 000 (USD142.9), per month, with 62% spending less than R1 000 (USD71.4) on groceries per person/month.

**Table 1:** Participants’ characteristics

	Overall	Urban		Rural	
Variables		Langa 1^a^	Langa 2^b^	Mt Frere^c^	*p*-value^g^
*N*	535	228	190	117	
Gender					
Women	411 (76.8)	175 (76.8)	140 (73.7)	96 (82.1)	0.241
Men	124 (23.2)	53 (23.2)	50 (26.2)	21 (17.9)	
Age					
< 50 years	264 (49.3)	75 (32.9)	161 (84.7)	28 (23.9)	0.001
≥ 50 years	271 (50.7)	153 (67.1)	29 (15.3)	89 (76.1)	
Education					
None or Primary	175 (32.7)	82 (36.0)	39 (20.5)	54 (46.2)	0.001
Secondary /Post-secondary School	360 (67.3)	146 (64.0)	151 (79.5)	63 (53.8)	
Employment					
Unemployed	410 (76.6)	175 (76.8)	131 (68.9)	104 (88.9)	0.001
Employed	125 (23.4)	53 (23.2)	59 (31.1)	13 (11.1)	
Household monthly income:					
< R2000	293 (54.8)	137 (60.0)	71 (37.4) ^h^	85 (72.6) ^h^	0.001
R2000–5000	193 (36.0)	75 (32.9)	89 (46.8) ^h^	29 (24.8)	
R5001–15000	49 (9.2)	16 (17.1)	30 (15.8)	3 (2.6)	
Monthly grocery expense/person					
< R1000	332 (62.1)	156 (68.4)	101 (53.2)	75 (64.1)	0.005
R1000–3500^e^	203 (37.9)	72 (31.6)	89 (46.8)	42 (35.9)	
Amount spent on groceries); mean (SD)	856.4 (19.9)	803.1 (28.0)	939.5 (36.4)	825.3 (42.0)	0.008
Grow vegetable/fruits in own garden (Yes)	95 (17.8)	9 (3.9)	0 (0)	86 (73.5)	0.001
Purchase fruits and vegetables daily/weekly (Yes)	379 (70.8)	175 (76.8)	17 (91.1)	31 (26.5)	0.001
Buy sugary drinks daily/weekly	268 (50.1)	131 (57.5)	119 (62.6)	18 (15.4)	0.001
Buy sugary drink monthly/seldom	267 (49.9)	97 (42.5)	71 (37.4)	99 (84.6)	
Own a car					
Yes	38 (7.1)	19 (8.3)	11 (5.8)	8 (6.8)	0.597
No	457 (92.9)	209 (9.7)	179 (94.2)	116 (93.2)	
Own TV					
Yes	460 (86.0)	213 (93.4)	156 (82.1)	91 (77.8)	0.001
No	75 (14.0)	15 (6.6)	34 (17.9)	26 (22.2)	
Own functional fridge					
Yes	118 (22.1)	22 (9.6)	58 (30.5)	38 (32.5)	0.001
No	417 (77.9)	206 (90.4)	132 (69.5)	79 (67.5)	
Transportation to grocery store by					
taxi/bus/train	279 (52.1)	125 (54.8)	71 (37.4) ^i^	83 (70.9) ^i^	0.001
own vehicle	45 (8.4)	12 (5.3)	6 (3.2)	7 (6.0)	
Walking	211 (39.4)	91 (39.9)	113(59.5) ^i^	27 (23.1) ^i^	
Minutes walked to grocery store^f^					
> 15 min	140 (26.2)	97 (42.5)	13 (6.8)	30 (25.6)	0.001
1–15 min	395 (73.8)	131 (57.5)	177 (93.2)	87 (74.4)	
Diagnosed with diabetes					
Yes	129 (24.1)	108 (47.4)	4 (2.1)	17 (14.5)	0.001
No	406 (75.9)	120 (52.6)	186 (97.9)	100 (85.5)	
Diagnosed with hypertension					
Yes	126 (23.6)	44 (19.3)	22 (11.6)	60 (51.3)	0.001
No	409 (76.4)	184 (80.7)	168 (88.4)	57 (48.7)	
Had NCD ^d^	202 (37.8)	112 (49.1)	24 (12.6)	66 (56.4)	0.001
Had no NCD	333 (62.2)	116 (50.9)	166 (87.4)	51 (43.6)	

*PURE* Prospective Urban and Rural Epidemiology, *SD* standard deviation; Proportions are given as column percentages

^a^ Langa 1 (Main Langa: Moderate-High SES area- PURE study Cohort), ^b^ Langa 2 (low SES adjoining area– non-PURE study cohort); ^c^ Mt. Frere Rural (PURE study rural cohorts); ^d^ Reported any of diabetes, hypertension, heart disease, or stroke)

^e^ Three persons spent more than R3500 on groceries per month (and were added to this category)

^f^ Grocery store included a Spaza shop, retail shop, and convenience store in or outside the study community

^g^The mean difference is significant at 0.05 level (95% CI) based on the observed mean

^h–i^ Post-hoc analyses showed significant difference taking Income ^h^’R5001–15000′, and ^i^ ‘Own Vehicle’ as references

The average monthly household expenditure on groceries, including fruits and vegetables was R856 (USD61). In the urban low SES area, the monthly amount spent on groceries was higher compared to the moderately-high SES urban, and the rural areas (i.e. R940 vs. R803, and R825). About one-fifth (22%) had reported owning a functional refrigerator, 71% of the sample purchased fruits and vegetables on a weekly or daily basis, with only 26% in the rural community compared to 90% in urban (low SES area). Eighteen percent of the study sample had reported growing fruits and vegetables in their own gardens, and 50% of the participants had indicated purchasing SSBs on a daily or weekly basis. Access to the grocery store was mainly by taxi/bus or by walking, with 74% of the participants taking not less than 15 min to walk to a nearby grocery store.

**Fig. 2:**
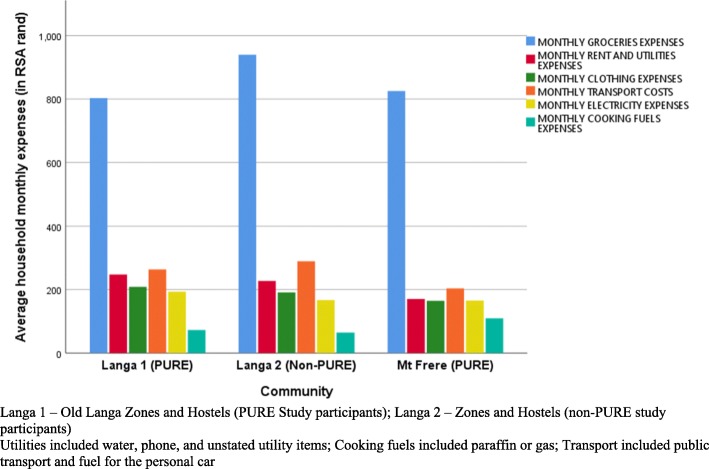
Household monthly expenses (on grocery, rent and utilities, transport, cooking fuel, and electricity) by community SES area

The average monthly household expenditure in South African Rand (ZAR) for basic household expenses is presented in Fig. 2. Nearly 84.5% of the monthly households’ income in each SES area was spent on groceries and the least on cooking fuel. The average amount spent on groceries per month was significantly higher than that spent on other household items and utilities (rent, transport, cooking fuel, and electricity) together; *p* = 0.009.

### Daily fruits and vegetables intake by socioeconomic status and morbidity

None of the study participants had reported consuming the WHO recommended daily 5-portions of fruits/vegetables at the time of the study. The daily intake of fruits and vegetables based on SES characteristics and self-reported chronic disease morbidity is presented in Table 2. Only 37.8% of the study sample consumed at least a portion of commonly available fruits and vegetables daily. There were no significant differences between those who consumed at least a portion of two fruits and two vegetables (and those who do not) by gender, household income categories, and community SES area. There was, however, significant differences in the mean ages between the two groups. The proportion reporting daily intake of at least two portions of fruits or vegetables was lowest in the rural community compared to the urban township (23.3% vs. 37.6 and 39.1%), although this was not statistically significant. However, significant differences between car ownership (*p* = 0.038), household expenditure on cooking fuel (*p* = 0.004), SSBs purchase (*p* = 0.001), and being diagnosed with diabetes (*p* = 0.016) were observed among those who consumed at least a portion of fruits and vegetables and those who do not. Purchase of SSBs was associated with intake of fruits and vegetables (*p* = 0.014). Specifically, the majority (57.5%) of those who had purchased SSBs daily or weekly had not consumed one or more portions of vegetables daily. Interestingly, average monthly income (which ranged between R200 and R15 000) had no significant association with daily intake of two or more portions of fruits/vegetables in the study sample. This, the authors believed that the later finding was due to the high poverty rate in these communities, as only a very small proportion (< 10%) of the study sample had reported employment/earning income monthly. In this population, monthly income/household is grossly low, and in the study, only 2.6% of the participants’ households had a monthly income of R5000 (US$357.1). Additionally, a higher proportion of those diagnosed with diabetes mellitus had not reported intake of one or more portions of fruit and vegetables daily.

**Table 2:** Daily intake of fruit and vegetables (proportions) by socio-economic status and reported NCD morbidity

Characteristics	Consumed at least a portion of commonly available fruits and vegetables daily ^b^		
	Yes	No	*p*-value*
N	202	333	
Socio-economic status (SES)	n (%)	n (%)	
*Sex*			
Men	46 (22.8)	78 (23.4)	0.475
Women	156 (77.2)	255 (76.6)	
*Overall*	202 (37.8)^a^	333 (62.2) ^a^	
*Age* (mean, SD)	51.1 (12.7)	48.8 (13.2)	0.055
*Community*			
Langa 1 (Urban, PURE cohort) ^c^	79 (39.1)	149 (44.7)	0.442
Langa 2 (Urban, non-PURE cohort) ^d^	76 (37.6)	114 (34.3)	
Mt. Frere (Rural, PURE cohort) ^3^	47 (23.3)	70 (21.0)	
*Household monthly income*			
< R2000	111 (55.0)	182 (54.7)	0.989
R2000–5000	73 (36.1)	120 (36.0)	
R5001–15000	18 (8.9)	31 (9.3)	
*Own a Fridge* (Yes)	50 (24.8)	68 (20.4)	0.144
*Own a Car* (Yes)	20 (10.0)	18 (5.4)	**0.038**
*Average monthly household expenditure* (in ZAR, mean, SEM^b^)			
Groceries	897.8 (13.3)	830.8 (14.9)	0.105
Rent and utilities	209.1 (11.9)	232.1(12.5)	0.132
Cooking fuel	91.9 (3.1)	69.1(3.8)	**0.004**
Clothing	196.8 (13.1)	190.1 (10.2)	0.688
Transport	254.8.2 (9.0)	262.3.7 (12.3)	0.713
Electricity	195.7 (10.9)	166.7 (8.5)	**0.012**
*Purchased sugar-sweetened beverages (SSBs)*			
Daily/Weekly	114 (42.5)	154 (57.5)	**0.014**
Monthly or seldom	88 (33.0)	179 (67.0)	
Morbidity			
Diabetes mellitus only (Yes)	38 (18.8)	91 (45.4)	**0.016**
Any NCD (diabetes, hypertension, heart disease or stroke)	70 (34.7)	132 (65.3)	0.144
Co-morbidity - two or more NCD (Yes)	23 (11.4)	38 (18.8)	0.556

Proportions are present as column percentages; ^a^ row percentage (overall Total)

*NCD* non-communicable diseases, *SD* standard deviation, *PURE* prospective urban and rural epidemiology, *SEM* standard error of mean

*NCD* Non-communicable diseases, *SEM* Standard error of mean

^b^ Those who reported eating at least two types (varieties) of fruit and/or vegetables

* *P*-values show comparisons at 95% CI: using Pearson Chi-square test for categorical variables and ANOVA test for mean comparison of continuous variables;

^c^ Langa 1 (Main Langa: Moderate SES area- PURE study Cohort), ^d^ Langa 2 (the Zones/low SES areas – non-PURE study cohort); Mt. Frere 1: Rural low SES – PURE study cohorts)

### Food items, fruits, and vegetables purchased by communities

The pattern of purchase of fruits and vegetables, and other selected food items by rural-urban location are presented in Table 3. More than half of the participants in the rural community had purchased fruits (56%) and vegetables (51%) either daily or weekly, and this was higher in the urban – fruits (93%) and vegetables (62%). However, significantly higher proportions (*p* < 0.05) of persons in the urban compared to the rural community had purchased SSBs (60% vs. 15%), snacks (61% vs. 15%) and sugar (33% vs. 3%) daily or weekly. Cereals, starchy food (maize meal, rice, flour, and pasta) and meat were mostly purchased monthly in both communities.

**Table 3:** Patterns of purchase selected food, vegetables and fruits by communities ^a^

	Selected food items, vegetables and fruits purchased^b^																	
	Cereals^c^		Starch ^d^		Meat ^e^		Vegetables ^f^		Fruit ^g^		Bread ^h^		SSBs^i^		Snack^j^		Sugar^k^	
	Urban	Rural	Urban	Rural	Urban	Rural	Urban	Rural	Urban	Rural	Urban	Rural	Urban	Rural	Urban	Rural	Urban	Rural
	n (%)^l^																	
Daily/Weekly	117 (28.0)	14 (12.0)	54 (12.9)	7 (6.0)	163 (39.0)	43 (36.8)	257 (61.5)	65 (55.6)	390 (93.39)	60 (51.3)	377 (90.2)	96 (82.1)	250 (59.8)	18 (15.4)	253 (60.5)	18 (15.4)	136 (32.5)	3 (2.6)
Monthly+	301 (72.0)	103 (88.0)	364 (87.1)	110 (96.0)	255 (61.0)	74 (63.2)	161 (38.0)	52 (44.4)	28 (6.7)	57 (48.7)	41 (9.8)	21 (17.9)	168 (40.2)	99 (84.6)	165 **(39.5)**	99 (84.6)	282 **(67.5)**	114 (97.4)
*p*-value**	**0.001**		**0.015**		0.747		0.285		**0.001**		**0.021**		**0.001**		0.001		**0.001**	

^**a**^ Urban Township (Langa) and Rural (Mount Frere) Community

^b^ Food items, vegetables and fruits purchased were reported in numbers (and frequency) – n(%)

^c^ – Cereals – oats, corn flakes, Weetabix, bran flakes, etc.;

^d^ – Starch – maize meal, samp (grounded maize), rice, flour, and pasta

^e^ – Meat – chicken, beef, mutton, pork, sausage;

^f^ – Vegetables – cabbage, spinach, pumpkin, carrots

^g^ – Fruit – Apple, pears, oranges, grapes, mango, avocado, banana;

^h^ – Bread – brown, white and whole wheat

^i^ – Sugar-sweetened beverages (SSBs) – 100% fruit juice, fizzy drinks, /Soft drinks;

^j^ – Snacks –Potato chips, peanuts, candies, biscuit, cakes

^k^ – Sugar – white or brown granulated sugar

^l^ - Number and proportion of respondents that purchase food items and fruits/vegetables daily/weekly and monthly

** *p*-value is at 95% confidence interval, and are reported for the comparison of purchases (daily to monthly) between the urban and rural communities. Bolded *p*-values are those comparisions that are statistically significant

In Table 4, the consumption patterns (count, and frequencies) of the selected commonly available fruits and vegetables in the two study communities are compared. Generally, the proportions of individuals who consumed each type of fruits and vegetables (except for onions) daily were comparatively lower in the rural community compared to the urban township. Invariably, the selected fruits and vegetables were consumed most often on a weekly basis, with apples, carrot, spinach, pumpkin, and cabbage being the most consumed. Low fruits consumption was seen in fruits like oranges were consumed (33%) in the rural, and similarly, pears (35%) and peach (42%) in the rural community – consumed only on monthly basis.

**Table 4:** The frequency of intake of selected commonly available fruits and vegetables by community

	Daily	Weekly	Monthly	Seldom	*p*-value*
Fruit					
Oranges	n (%)				
Urban	87 (20.8)	261 (62.4)	35 (8.4)	35 (8.4)	0.001
Rural	20 (17.1)	38 (32.5)	39 (33.3)	20 (17.1)	
Apples					
Urban	81 (19.4)	280 (67.0)	38 (9.1)	19 (4.5)	0.001
Rural	18 (15.4)	44 (37.6)	37 (31.6)	18 (16.4)	
Pears					
Urban	58 (13.9)	218 (52.2)	87 (20.8)	55 (13.1)	0.005
Rural	9 (7.7)	30 (25.6)	37 (31.6)	41 (35.0)	
Banana					
Urban	81 (19.4)	258 (61.7)	51 (12.2)	28 (6.7)	0.001
Rural	15 (12.8)	42 (35.9)	39 (33.3)	21 (17.9)	
Peach					
Urban	77 (18.4)	219 (52.4)	50 (12.0)	72 (17.2)	0.003
Rural	4 (3.4)	19 (16.2)	45 (38.5)	49 (41.9)	
Vegetables					
Tomato					
Urban	61 (14.6)	273 (65.3)	50 (12.0)	34 (8.1)	0.001
Rural	33 (28.2)	48 (41.0)	23 (19.7)	13 (11.1)	
Onions					
Urban	155 (37.1)	212 (50.7)	50 (12.0)	1 (0.2)	0.005
Rural	73 (62.4)	22 (18.8)	20 (17.1)	2 (1.7)	
Spinach					
Urban	34 (8.1)	324 (77.5)	57 (13.6)	3 (0.7)	0.012
Rural	15 (12.8)	58 (49.6)	38 (32.5)	6 (5.1)	
Cabbage					
Urban	29 (6.9)	335 (80.1)	48 (11.5)	6 (1.4)	0.001
Rural	38 (32.5)	50 (42.7)	26 (22.2)	3 (2.6)	
Pumpkin					
Urban	18 (4.3)	322 (77.7)	76 (18.2)	2 (0.5)	0.001
Rural	13 (11.1)	39 (33.3)	52 (44.4)	13 (11.1)	
Carrot					
Urban	65 (15.6)	292 (69.9)	59 (14.1)	2 (0.5)	0.001
Rural	47 (40.2)	33 (28.2)	29 (24.8)	8 (6.8)	

Proportions are presented as row percentages

* *p*-value is at 95% confidence interval, and is based on the Pearson Chi-square comparison between the two communities for the categorical variables

Further analyses showed some differences in the consumption of either fruits or vegetables by SES area, as seen in Table 5. Very low intake of at least two types of each of fruits and vegetables was reported (13%), with a high proportion in the rural compared to urban sites (27% vs. 10% vs. 9%).

**Table 5:** Patterns of daily intake of commonly available fruits and vegetables by community area/site

Daily fruits and vegetables intake^a^	Total	Langa 1 (PURE)	Langa 2 (non-PURE)	Mt Frere (rural)	*p*-value^b^
Two portions of fruits and or vegetables daily	202 (37.8)	79 (34.6)	76 (40.0)	47 (40.2)	0.442
One or more portions of fruit daily	193 (36.1)	82 (36.1)	81 (42.6)^c^	30 (25.6)	0.011
One or more portions of vegetables daily	255 (47.7)	109 (47.8)	67 (35.3)	79 (67.5)^c^	0.001
Eaten fruit and vegetables (at least two of each) daily	71 (13.3)	20 (8.8)	19 (10.0)	32 (27.4)	0.001

Langa 1 – Old Langa Zones and Hostels (comparatively moderate SES); Langa 2 – Zones and Hostels (Comparatively **low** SES area); Mt. Frere (typical rural economically disadvantaged – Poor SES)

*PURE* Prospective Urban and Rural Epidemiology Study

^a^ A portion of fruit (i.e. one half to 1 large/small size of commonly available fruits), and a portion of vegetable, as one half to one cup of the listed vegetables);

^b^ *P*-value is at 95% confidence Interval (CI)

^c^ Post-hoc analysis was significant

### Determinants of daily fruits and vegetables intake

Multivariate regression model (Table 6) indicated that those who spent R1 000 (USD71.4) and more on groceries per month were 1.6 times (AOR 95% CI, 1.05–2.44; *p* = 0.030) more likely to eat fruits and vegetables (two portions or more) daily compared to those who spent less. In addition, those who travelled with a personal vehicle from home to purchase groceries were 2.1 times (AOR 95% CI, 1.06–4.09; *p* = 0.003) more likely to eat fruits and vegetables compared to those who had walked to purchase groceries. Education, monthly household income, owning a fridge (for storage), and being diagnosed with a chronic condition (diabetes and hypertension) had no significant association with daily fruits and vegetables intake, whereas purchasing SSBs daily or weekly had an inverse association with daily intake of fruits and vegetables.

**Table 6:** Factors associated with daily intake of two portions of commonly available fruits and vegetables

	Univariate analyses			Multivariate logistic regression		
	Odds ratio	95% CI	*p*-value	Odds ratio	95% CI	*p*-value
Variables						
Socio-demographics						
Gender (Female)	2.50	0.24–4.02	0.056	1.03	0.66–1.59	0.900
Age (≥50 years)	0.78	0.48–1.08	0.856	1.58	1.01–2.42	0.028
Community (urban)	2.95	1.03–4.56	0.070	1.53	0.88–2.67	0.132
Socio-economics						
Household monthly income						
<R2000 (ref)						
R2000–5000	0.75	0.48–1.16	0.199	0.75	0.48–1.16	0.199
R5001–15000	0.68	0.33–1.39	0.291	0.68	0.33–1.39	0.291
Own a refrigerator (Yes)	1.28	0.84–1.94	0.251	1.01	0.64–1.60	0.962
Monthly grocery expenditure (≥R1000)	1.56	1.05–2.44	0.005	1.60	1.05–2.44	**0.030**
Attitude						
Perceived fruits and vegetables as healthy food (Yes)	0.82	0.53–1.27	0.37	0.78	0.49–1.24	0.290
Food choice and access						
Buy fruits/vegetables daily or weekly (Yes)	1.119	0.74–1.69	0.067	1.119	0.74–1.69	0.596
Travel to purchase groceries by:						
Taxi, bus, or train (ref)						
Personal vehicle	1.08	1.06–3.09	0.005	2.084	1.06–4.09	**0.003**
Walk	2.66	1.06–4.09	0.040	1.524	0.97–2.40	0.069
Purchased SSB daily or weekly (Yes)	0.54	0.36–0.81	0.008	0.541	0.36–.810	**0.007**
Morbidity						
Diagnosed with diabetes (Yes)	1.76	1.44–2.30	0.005	0.755	0.44–1.30	0.311

## Discussion

Adequate intake of fruits and vegetables is considered an essential option for disease prevention and maintaining optimal health, our study points to low purchase and inadequate daily intake in these communities. There were disparities in the purchases and consumption patterns of fruits and vegetables by rural-urban location. There were variations in the commonly purchased fruits and vegetables by location. Daily consumption of fruits and vegetables were also low particularly in the rural community compared to the urban.

The analysis also showed that those who had spent R1 000 (USD71.4) or more per month on groceries were mostly like to consume a portion of fruits and vegetables daily. In contrast, those who consumed SSBs daily/weekly were significantly less likely to consume fruits and vegetables daily. These results are discussed in details.

### Low purchase and daily intake of fruits and vegetables.

This study has shown that the amount spent on monthly grocery purchases and transport was highest in the township zones/hostels with the least socio-economic status confirming previous studies in the general population of South Africa [1]. In addition, patterns of purchase and daily consumption of fruits and vegetables were lowest in the rural and poor urban area, even though this setting seems to be producing the fruits and vegetables for the urban population. These findings point to the challenge of equity and food insecurity in disadvantaged settings and may inform sustainable intervention on food security.

In addition, the urban community purchased fruits and vegetables most often on a weekly basis, whereas in the rural community a substantial proportion (~ 40%) purchased fruits and vegetables monthly or seldomly. This underlying pattern of fruits and vegetable purchases may be linked to their high cost often reported in poor South African settings [1, 21, 32]. This study confirms the previous reports that indicated a low intake of fruits and vegetables reported in many South African settings, particularly in the rural and urban poor communities^(^[1, 3, 23]^)^.

The national survey (SANHANES-1) conducted in 2012 also reported a low intake of fruit and vegetables of two or fewer portions per day in about a third of South Africans^(^[15]^)^. Spending more than R1000/month and having personal transport to purchase groceries were key determinants of intake of daily fruits and vegetables. However, a study of non-African populations had shown that determinants of low consumption of fruits and vegetables were: perception on affordability, and absence of financial means to buy fruits and vegetables daily, younger age (< 55 years), and education level lower than tertiary [33]. The inverse association of daily/weekly SSBs intake with daily consumption of fruits and vegetables reported in this study is of considerable importance. The increasing access to readily available and cheaper SSBs can lead to substitution of fruits and vegetables (which are often costly) with SSBs for in economically disadvantaged communities [34]. Although inadequate fruits and vegetable consumption is a problem worldwide [35], the situation in South Africa is of critical concern, as most people in the disadvantaged communities do not have access to farmland to produce their fruits and vegetables. Also, poor access to land has made farming and gardening difficult in the disadvantaged communities, compounding the problem of food insecurity in this setting [36]. The impact of the increasing access to cheap SSBs on fruits and vegetables intake was reported in a previous study [21]. Also, a recent study by Okop et al. [22] had reported that persons from food-insecure South African households in two selected communities had consumed more SSBs servings per week than the food-secure ones, and this was also associated with weight gain. Notably, vegetable consumption among South Africans, for instance, had decreased by about 8.0% between 1999 and 2012 [21]. Moreover, in these impoverished communities with high unemployment, the income level of the study participants had no significant association on the daily intake of fruits and vegetables. Having no income, being unemployed with limited social agencies, place many in a situation with no choice for healthy nutrition, as striving to survival (‘striving to eat anything to fill the stomach’) will be the ultimate goal.

Our findings also show that there was no significant association between owning a functional refrigerator and daily intake of fruit and vegetables. This could likely mean that even though people may have refrigerators to store fruits and vegetables, they may not have enough to last longer for sustained daily consumption. Besides storage, the effect of seasonality on the intake of fruits and vegetables could also affect the pattern of purchase and consumption. Improving purchase by reducing prices (through subsidies), increasing access to fresh fruits and vegetables (through incentivising with coupons system) [37] could probably increase purchase and invariably, daily intake considerably according to Temple et al. [34]. Communal or shared-refrigeration at the community level can be supported to enhance preservation of fruits and vegetables for a longer period, and to avoid daily purchase. Institutional policies on fruits and vegetables for health reasons should include strategic school and worksite feeding programmes that support adequate nutritious food.

Summarily, this study had shown that the amount spent on monthly grocery purchases and transport was highest in the township zones/hostels with the least socio-economic status confirming previous studies in the general population of South Africa. In addition, patterns of purchase and daily consumption of fruits and vegetables were lowest in the rural and poor urban area. These findings point to the challenge of equity and food insecurity in disadvantaged settings and may inform sustainable intervention on food security.

### Impact of affordability and purchase of SSBs and snacks

This study showed that substantially high proportions of those living in socio-economically disadvantaged communities (based on our sampled population), particularly, the urban township had purchased sugary drinks daily/weekly and had spent a substantial amount of their monthly household income on groceries, utilities and transport. Moreover, the average monthly household expenditure on groceries (including fruits and vegetables) was significantly higher than the expenditure on other household items and utilities put together, even though less than 3% of households earned US$357 (R5000)/month. Moreover, in the two study communities, only very few households (17.8%) own gardens where they could produce fruits and vegetables for their consumption. The urban informal low SES areas had no one who owns a garden or is growing fruits or vegetables for personal consumption. These findings point to economic deprivation due to lack of jobs, employment, and access to land for cultivation by the disadvantaged black South Africans.

In addition, purchasing and consuming fruits and vegetables daily or weekly tended to be lowest in the rural and poor urban areas, as previously reported in poor South African settings, perhaps, due to poverty and food insecurity [21, 38]. It could be implied that, although the study participants had access to the commonly available fruits and vegetables, purchasing and consuming these needed some agencies to be actualized. Findings from recent study in Cape Town collaborated this assertion, as it showed that persons living in poor-resource areas (usually the food insecure persons) have poor purchasing power and purchased fruits and vegetables less frequently [18]. Also, these persons purchased more of less expensive SSBs and snack items more frequently than those in high-income areas. Moreover, the formative evidence from a 4.5 year longitudinal cohort, including 800 adults in South Africa (SA), from the harmonised STOP-SA study indicated that: i) the socio-economically disadvantaged (mainly the food-insecure) persons are more likely to purchase SSBs and salty snacks, and less likely to purchase vegetables/fruits, than those that are food secure, and ii) high intake of SSBs, and low intake of vegetables/fruits predicts weight gain over this period [16, 17].

In summary, these findings could imply that due to very low and competing needs for utilities and transport, and with the little means for choice, dietary preference for sugary beverages and snacks food might be one main choice. This assertion is collaborated by previous studies which have shown that access to cheap sugary drinks can impact negatively on the daily intake of fruits and vegetables in this setting [16, 39]. In contrast, a study conducted in 7 Asian countries has shown that the use of substances such as alcohol had no significant influence on the poor intake of vegetables and fruits [40].

Furthermore, our study showed that a higher proportion of those who reported having diabetes mellitus did not consume a portion of two fruits and vegetables daily. This finding has implications for the management of patients with diabetes in poor communities. Moreover, the intake of a diet deficient in fruits and vegetables and other nutrients is linked with increasing NCD [41].

The issues of cost, availability and access to healthy food are considered key factors that influence the purchase and consumption of these foods. In this study, those living in economically disadvantaged areas (with low income and high unemployment) spent more on groceries - mainly fruits and vegetables. This finding confirms the findings from recent and previous studies indicating that persons living in low-income households or settings spend more on fruits and vegetables [3, 16]. In addition, those who could afford R1 000 groceries per month were about two times more likely to consume at least two portions of fruits and vegetables. According to Temple et al., a healthier diet is largely unaffordable for most South Africans, as this can cost as much as 69% more than a typical South African diet [34]. Taste, health, nutrient content, safety and quality, and ease of preparation are considered after the price of food [42].

### Promoting adequate fruits and vegetables consumption

The challenging food environments and the increasing retail supermarkets in economically disadvantaged communities, unfortunately, provide easy access to available and relatively cheaper, high-calorie ‘obesogenic’ food and SSBs [43, 44]. The above challenge and the high level of inequality, poverty and unemployment might be responsible for the low intake of fruits and vegetables among those living in poor settings. These have been reported to often purchase cheaper and affordable high calorie unhealthy (obesogenic) foods perhaps due to cost and access [21]. Intake of these obesogenic foods, particularly, SSBs and snacks were recently linked to relative weight gain in this same study population [22]. As indicated by our study, affordability and access to SSBs are considered important factors that can affect adequate fruits and vegetables intake patterns among the poor. Interventions that can promote sustainable production and access to affordable varieties of fruit and vegetables should, therefore, be implemented in resource-poor communities.

### Strategic interventions are needed

Strategic interventions that promote sustainable access to affordable fruits and vegetables and discourages the aggressive promotion of SSBs in the communities is therefore critical to addressing the problem of low intake. It is envisaged that this will mitigate the health impact of low intake of fruits and vegetables in the resource-poor populations. There is the need to build the capacities of Stokvel (an example of rotating savings and credit associations – ROCSA) highly prevalent in South African communities to create demand for healthy food in the communities. This can be achieved, if Stokvels, such as grocery or saving stokvel groups harness their resources to buy bulk at the source and deliver to their members, and create awareness on universal accesses to healthy food. Furthermore, awareness campaigns on the health benefits of fruits and vegetables consumption should be undertaken and target meso-level actors in the food environment such as community leaders, consumers in the households and schools, and food producers and handlers. These set of actors can be reached through community-based health promotion programmes targeting specific food players in the communities. In addition, a combining subsidies on healthy foods and taxes on unhealthy foods which has been shown to be cost-effective in improving diet and population-health should be commissioned by the government [45]. Finally, policies that seek to promote subsidies on healthy food should be implemented in the Provinces to bring about sustainable access to affordable fruits and vegetables particularly.

### Strengths and limitations of the study

We obtained our data from two South African economically disadvantaged communities which allows comparison of food purchasing and consumption patterns among poor communities in two settings. Generally, the two communities had low monthly household income and high (77%) unemployment rates which might have impacted on the purchasing power. Many of the types of fruits and vegetables were also purchased on a monthly or weekly basis, leading to a higher weekly/monthly consumption but low daily consumption as suggested in the food-based dietary guidelines.

This study has some limitations. The quantified dietary data were not collected making it impossible to report on the average daily amount of fruits and vegetables consumed per person. Also, in our multivariate logistic model, we did not totally control for all expected confounders such as price of and access to fruits and vegetables, food insecurity, smoking status, and number of children in the household which could influence intake of fruits and vegetables. However, we had controlled for sociodemographic factors (income, gender, age, and location), monthly expenditure on groceries, consumption of other foods (SSBs), travel to buy food, and attitudes (or preference) of fruits and vegetables as healthy food. In addition, the study participants were predominantly women, most of them unemployed, with no minimal household income, which therefore could contribute some possible bias. The skewed gender proportion could contribute a bias to the study; however, this was controlled for in our final model. Furthermore, this is a cross-sectional study, and can only allow us to determine the associations between outcome variable and explanatory variables. The findings can be applied to the poor-resource settings in South Africa and other African populations. Future studies should use larger more representative populations to determine and compare the impact of socioeconomic status (low, medium, high) on fruits and vegetables consumption in the entire population of South Africa. Future research should also consider the effect of seasonality, food insecurity on daily intake of fruits and vegetables in resource-poor settings.

## Conclusion

This study has shown that affordability and frequency of purchase of sugary drinks can influence the daily intake of fruits and vegetables in resource-limited communities. Strategic interventions that can promote sustainable access to affordable quality fruits and vegetables are critically needed to address the problem of poor intake and to mitigate the health.

## Data Availability

All the data contained within the manuscript and the materials are available from the corresponding author upon request.

## Abbreviations

CoEFS: Centre of Excellence on Food Security
DALY: Disability-adjusted life years
NCDs: Non-communicable diseases
PURE Study: Prospective Urban and Rural Epidemiology Study
SANHANES: South African National Health and Nutrition Survey
SSBs: Sugar-sweetened beverages
